# Bibliometric and visual analysis of cerebral revascularization from 1999 to 2022

**DOI:** 10.3389/fnins.2022.1088448

**Published:** 2023-01-09

**Authors:** Ding Zhang, Xiaoqian Li, Ni Jia, Wei Chen, Yueqiang Hu

**Affiliations:** ^1^Guangxi University of Chinese Medicine, Nanning, China; ^2^Weinan Vocational and Technical College Nursing College, Weinan, China; ^3^Department of Encephalopathy, The First Affiliated Hospital of Shaanxi University of Traditional Chinese Medicine, Xi’an, China; ^4^Department of Encephalopathy, The First Affiliated Hospital of Guangxi University of Chinese Medicine, Nanning, China

**Keywords:** cerebral revascularization, VOSviewer, CiteSpace, bibliometric, visualization

## Abstract

**Background:**

Cerebral revascularization is a neurosurgical procedure used to restore the cerebral collateral circulation channel. This study examines the countries, institutions, authors, journals, keywords, and references related to the disease in the field of cerebral revascularization from 1999 to 2022 from a bibliometrics perspective, evaluates the changes of knowledge structure clustering and identifies the new hot spots and new research directions in this field.

**Methods:**

The Web of Science Core Collection (WOSCC) database and the PICOS retrieval method were used to conduct a comprehensive search for articles and reviews pertaining to cerebral revascularization. The final filtered data were bibliometrically and visually drawn using Microsoft office 365, CiteSpace (v.6.1.R2), and VOSviewer (v.1.6.18).

**Results:**

From 1999 to 2022, a total of 854 articles pertaining to cerebral revascularization, which originated from 46 nations, 482 institutions, and 686 researchers, were extracted from the WOSCC database, and the number of publications in this field of study was rising. The United States held the highest proportion in the ranking analysis of countries, institutions, authors, and journals. By analyzing co-citations, the scientific organization of this field and the development status of frontier fields were realized. Cerebral revascularization, moyamoya disease, extracranial intracranial bypass, and occlusion are the current research focal points in the field of cerebral revascularization. Hyperperfusion and vascular disorder may also become a new study focus in this discipline in the near future.

**Conclusion:**

Using the method of bibliometrics, this study analyzed and reviewed the articles in the field of cerebral revascularization, which enabled scholars to better comprehend the dynamic process in this field and provided a foundation for future in-depth research.

## 1. Introduction

Ischemic stroke is the third leading cause of mortality after cardiovascular disease and cancer and the leading cause of disability in the world, which is characterized by high incidence and high lethality (Ding et al., 2022). Carotid atherosclerotic stenosis is a prominent cause (Heck and Jost, 2021). According to researches, stroke remains the top cause of all-cause mortality, and ischemic stroke accounts for more than 80% of cases, with a substantially higher incidence than other forms of stroke and a recurrence rate that has exceeded 48%, gravely limiting the development of human health (Krishnamurthi et al., 2020; Skajaa et al., 2021; Syahrul et al., 2021). As human’s awareness of stroke diseases increase, the clinical treatments of ischemic stroke continue to evolve (Megaly et al., 2020). Injection of recombinant tissue-type fibrinogen activator (rt-PA) is the most effective clinical treatment for cerebral ischemia (Zi et al., 2021). However, rt-PA is less effective in treating chronic cerebral ischemia diseases, such as moyamoya disease, cerebral atherosclerosis and carotid artery stenosis/occlusion (Wang et al., 2020). Consequently, the treatment of such refractory cerebrovascular illnesses has gradually become the focus of study in a number of countries. Several clinical studies have demonstrated that carotid endarterectomy (CEA) and carotid stenting (CAS) are significantly superior to optimal drug therapy for treating carotid artery stenosis and preventing stroke recurrence and death (Dharmakidari et al., 2017; Lamanna et al., 2019; Halliday et al., 2021), whereas carotid occlusion is a condition in which blood flow is completely obstructed as luminal stenosis increases due to atherosclerotic lesions. The etiology of ischemic stroke caused by carotid blockage includes not only distal thromboembolism but also hemodynamic disruption, or, even worse, the presence of both risk factors (Cagnazzo et al., 2020).

In recent years, with the continuous in-depth study of imaging technology and clinical understanding of carotid artery occlusion, an increasing number of clinical researchers have gradually realized that hemodynamic disorders play a crucial role in cerebral ischemia caused by carotid artery occlusion (Tsukada et al., 2022). The study revealed that among patients with carotid artery occlusion receiving drug treatment (Li et al., 2020, 2022), the annual incidence of ipsilateral ischemic stroke was 3%, but the annual incidence of stroke increased to 10–20% in patients with severe cerebral hemodynamic disorders. The enhancement of brain hemodynamics has therefore received considerable attention from researchers.

With the continuous development of science and technology, procedures related to cerebral revascularization have taken a place in chronic refractory ischemic cerebrovascular diseases (Rennert et al., 2019). Since the first successful clinical application of intracranial-extracranial vascular bypass (EC-IC) in the world, revascularization surgery for cerebrovascular diseases has remained resilient despite the ups and downs (Arenillas et al., 2019), playing an irreplaceable role in the treatment of moyamoya disease, intracranial vascular malformations, and complex aneurysms (Gross and Sacho, 2021). With the ongoing appearance of new intravascular therapeutic materials and the advancement of cerebral revascularization technology, the intravascular recanalization technology for occluded arteries has become progressively developed (Hu et al., 2019). Currently, cerebral revascularization technology is experiencing a significant resurgence. Different types of revascularization for the treatment of ischemic cerebrovascular disorders have sparked substantial debate among academics around the globe (Lopez-Gonzalez et al., 2022). Nevertheless, there are no exhaustive research on the current status, hot spots and trends in the treatment of cerebrovascular disorders. Consequently, this article employs the bibliometric method and uses CiteSpace and VOSviewer, two recognized metrological tools, for analysis and statistics, in order to identify recent hot areas and trends in the research field of cerebral revascularization.

Bibliometrics is an effective way to explore the development track of a discipline. At the same time, it is also a discipline that analyzes the measurement characteristics, change rules and knowledge systems of literature and other communication media (Zhong and Lin, 2022). Bibliometrics originated in the field of library and intelligence. In 1969, the British intelligence scientist Pritchard formally introduced “Bibliometrics,” which symbolized that it established as an independent discipline. However, with the progress of science and technology and the development of the times, information exchange has become more convenient, and the cross-connections and knowledge interaction between disciplines have become closer. At present, bibliometrics has been widely used in many fields such as information science, economic management, and biomedicine. Lin et al. (2020) analyzed the dynamic development process of Pythagorean fuzzy sets (PFSs) using bibliometrics, providing a new perspective for scholars in the field of PFSs to grasp the research hot spots. Based on a large number of literature studies, (Yu et al., 2021) conducted a comprehensive analysis of the Analytic hierarchy process (AHP) by using bibliometrics. In summary, we believe that bibliometrics can effectively evaluate the current development status and trends of a research field to uncover the potential knowledge value (Yu et al., 2019). Both in theory and in practice, it has played an important role in guiding the development of many disciplines.

CiteSpace is a mapping software created by Dr. Chaomei Chen for the visual study of voluminous amounts of literature, which can rapidly sort the growth history of a research topic and discover essential literature and major research teams (Luo and Lin, 2021; Chen et al., 2022). VOSviewer is a mapping application developed by Dr. Ness Jan van Eck and Dr. Ness Jan van Eck of Leiden University in the Netherlands for constructing and visualizing literature measurements. It is a free web application for constructing knowledge maps and viewing network graphs (Yu et al., 2020; Zhang and Lin, 2022). As a result, we have conducted a metrological analysis based on published studies in the field of cerebral revascularization research and are prepared to respond to the following questions: What is the production and distribution of science in this field? What are the most popular current research topics in this field? This study aims to disclose the distribution, hot themes and trends of research in the field of cerebral revascularization, and to give physicians the most recent data in this research area so that they can make informed judgments.

## 2. Materials and methods

### 2.1. Ethical review

Ethical review was not required for this study, because all the original data used here were from public databases and secondary literature analysis was performed on the data.

### 2.2. Data sources

**FIGURE 1:**
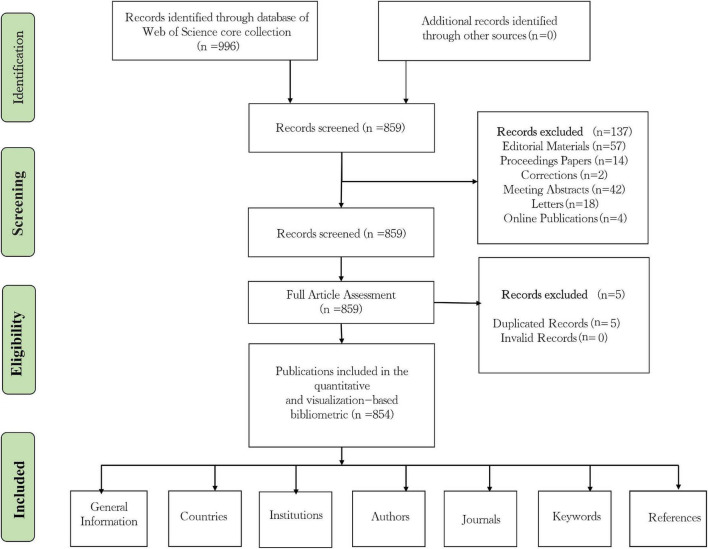
Document screening flow chart.

The Web of Science Core Collection (WOSCC) is the source of all data in this study since it is widely acknowledged as the most complete and high-quality digital literature database, as well as the most authoritative bibliometric analysis database. We combed through the WOSCC database from 1999 to 2022, and used “Cerebral Revascularization” (subject) or “Extracranial-Intracranial Arterial Bypass” (subject) or “Cerebral Microsurgical Revascularization” (subject) or “EC-IC Arterial Bypass” (subject) or “Bypass, STA-MCA” (subject) as the search phrase to retrieve relevant articles. English is only allowed as a search language. Eventually, 996 papers were retrieved in total, including 769 articles, 90 reviews, 14 conference papers, 57 editorial materials, 42 conference abstracts, 18 letters, 2 revisions, and 4 online publications. We restricted the scope of literature categories to papers and reviews, resulting in the collection of 859 papers. Two researchers examined 859 articles manually. In the event of a dispute, the third researcher has final say. In the end, five duplicate articles and zero invalid articles were removed, resulting in 854 articles. The specific flowchart is illustrated in Figure 1.

### 2.3. Data analysis

This study uses Microsoft Office 365, CiteSpace (v.6.1. R2), and VOSviewer (v.1.6.18) to conduct bibliometric and visual analysis of all data.

Use VOSviewer (v.1.6.18) to do bibliometric analysis on countries/regions, institutions, journals, authors, keywords, and references and build visual maps. Each node on the map indicates the respective country/region, institution, journal, author, keyword, and citation. The line between the nodes represents the relationship of collaboration. CiteSpace (v.6.1. R2) is utilized to construct a two-sided map coverage analysis of countries/regions, institutions, authors, key phrases, co-introduction references, and co-introduction journals. In the map, the size of nodes is proportional to the number of references, and the thickness of lines between nodes shows the strength of collaboration (Ma et al., 2020). CiteSpace (v.6.1. R2) software parameter settings are as follows: Time Slicing: From 1999 Sep to 2022 Sep, #Year Per Slice (1); Text Processing: Term Source includes Title, Abstract, Author Keywords (DE), and Keywords Plus (ID); Term Type, Links and Selection Criteria; Node Type: Author, Institution, Country, Term, Keyword, Source, Category, Reference, Cited Author, and Cited Journal; Pruning includes: Pathfinder, Pruning Sliced networks, Minimum Spanning Tree, and Pruning the merged network.

## 3. Results

### 3.1. General data

**FIGURE 2:**
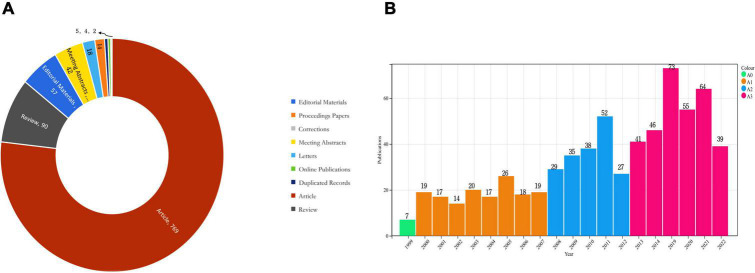
**(A)** Types of publications in the field of cerebral revascularization. **(B)** Trends in the volume of publications in the field of cerebral revascularization.

We deleted five duplicate items through manual screening. A search of the WOSCC database from 1999 to 2022 yielded a total of 854 articles related to cerebral revascularization. In addition, the quantitative analysis incorporated 46 nations, 482 institutions, and 689 scholars. The types of publications included are as follows: Figure 2A indicates that there were 769 papers, 90 reviews, 14 conference papers, 57 editorial materials, 42 conference abstracts, 18 letters, 2 revisions, and 4 online publications.

As shown in Figure 2B, we also analyzed the number of global publications on cerebral revascularization published annually. In general, the number of publications in this research field has increased. Especially since 2008, the number of publications in the field of cerebral revascularization has increased rapidly, with less fluctuations and an average of 40 to 50 publications from 2013 to 2022. Furthermore, it is evident that the low volume of research before 2008 may have been limited by various factors, such as insufficient equipment, immature personnel skills, and underdeveloped information technology. With the continuous development of science and technology, the number of studies in this field is increasing, which also highlights the current global medical field has been vigorously promoted by science and technology. At the same time, the development of cerebral revascularization has gradually attracted the attention of the public, and it has become a new direction in the field of neurology.

### 3.2. Distribution of countries and institutions

**FIGURE 3:**
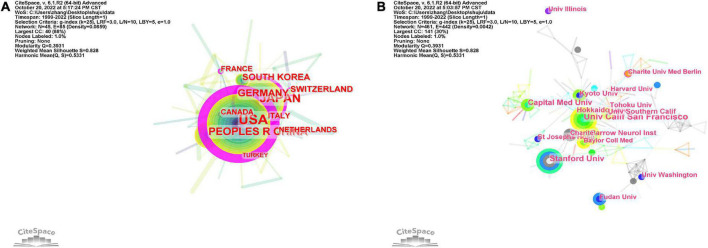
Analysis of countries and institutions. **(A)** Visual map of national collaboration based on CiteSpace software. **(B)** Visual map of institutions based on CiteSpace software. Each node represents a country/institution, and each connecting line represents the central cooperativeness between countries/institutions. The thicker the line, the closer the cooperation between countries/institutions.

By analyzing the original data, it was found that 482 institutions from 46 countries contributed content on cerebral revascularization. The United States has published the most publications (339), followed by Japan (173), China (99), Germany (56), South Korea (48), and Switzerland (38 articles) (Figure 3A and Table 1). It is not difficult to find that in the ranking of publishing countries, most are developed countries, especially dominated by the United States, with earlier scientific and technological development than other countries. However, as a big developing country, China is now close to the top three in the world in this field, which also shows that China has injected fresh blood into the research of cerebral revascularization and is the main country to promote the development of this field. The United States has the greatest central position, with a central threshold of 0.99, indicating a clear edge in terms of national collaboration and national influence (Table 2). Japan (0.19), the United Kingdom (0.15), Germany (0.12), Switzerland (0.11), Canada (0.1), and France (0.1) also have a prominent central position. Even though the document outputs of China and South Korea are among the top five in the world, there is no apparent advantage in the central position, with 0.09 and 0.05, respectively. This also demonstrates that a number of Asian nations continue to have deficiencies in national collaboration and national influence, but we cannot disregard their contributions to cerebral revascularization research. Of course, another important reason for China’s low index of national cooperation and national influence is the resource blockade and the isolation of cutting-edge technological talents in most capitalist countries. Therefore, we believe that national ideological differences cannot be applied to the cause of human health. China and the United States should uphold the spirit of cooperation, strengthen exchanges between various disciplines and the cultivation of talents, and jointly promote the forward development of the field of cerebral revascularization, and even the development of multidisciplinary fields.

**TABLE 1:** Ranking of countries in terms of publications (top 10).

Rank	Count	Centrality	Country
01	339	0.99	United States
02	173	0.19	Japan
03	99	0.05	China
04	56	0.12	Germany
05	48	0.09	South Korea
06	38	0.11	Switzerland
07	26	0.04	Italy
08	24	0.03	Netherlands
09	22	0.1	Canada
10	19	0.1	France

**TABLE 2:** Ranking of countries in terms of centrality (top 10).

Rank	Centrality	Count	Country
01	0.99	339	United States
02	0.19	173	Japan
03	0.15	10	United Kingdom
04	0.12	56	Germany
05	0.11	38	Switzerland
06	0.1	22	Canada
07	0.1	19	France
08	0.09	48	South Korea
09	0.05	99	China
10	0.04	26	Italy

In the distribution of national institutions (Figure 3B and Table 3), we found that the University of California, San Francisco, United States has the most articles (28), followed by Stanford University in California, United States (23), Capital Medical University of China (20), St. Joseph’s Hospital in Los Angeles, United States (18), the University of Southern California, United States (16), Northeastern University of Japan (15), and Barrow Institute for Medieval and Renaissance Studies (15). As expected, the major institutions and universities in the United States enjoy a distinct edge in terms of the number of documents granted. It reveals that the United States is the global leader in cerebral revascularization research. At the same time, in this field of research, the strength of Asian countries should not be underestimated, especially the major institutions and universities in China and Japan, which started late but developed rapidly and are the backbone of the field of cerebral revascularization. In addition, in the process of global economic integration, we should also call for close cooperation between institutions and universities in the field of cerebral revascularization for the sake of human health.

**TABLE 3:** Ranking of institutions with the largest number of publications (top 10).

Rank	Count	Centrality	Country	Institution
01	28	0.07	United States	University of California, San Francisco
02	23	0.01	United States	Stanford University
03	20	0.04	China	Capital Medical University
04	18	0.01	United States	Los Angeles St. Joseph’s Hospital
05	16	0.01	United States	University of Southern California
06	15	0.05	Japan	Tohoku University, Japan
07	15	0.07	United States	Barrow Institute of Neurology
08	15	0.02	Japan	Hokkaido Univ
09	14	0.01	United States	Univ Washington
10	13	0.04	Japan	Kyoto Univ

### 3.3. Distribution of authors and co-cited authors

**FIGURE 4:**
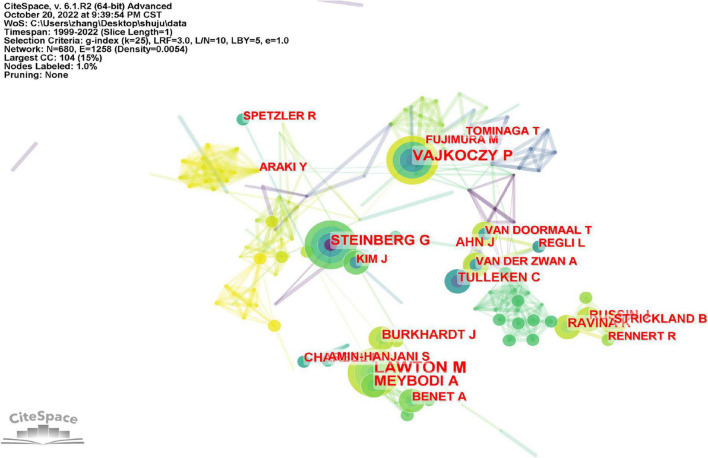
Analysis of authors published the most articles based on the CiteSpace visual map. Each node represents an author’s name, and each line represents the central collaboration between authors. The thicker the line, the closer the collaboration between the authors.

**FIGURE 5:**
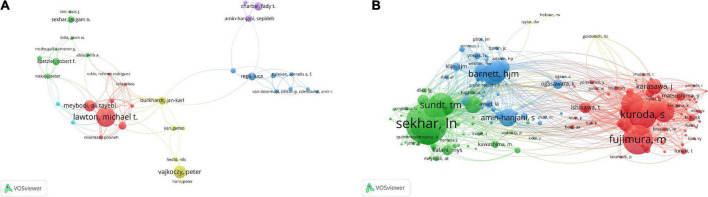
Author analysis. **(A)** Author collaborative network analysis based on VOSviewer visual map. **(B)** Co-citation author network analysis based on VOSviewer visual map. The clusters of different colors reflect the cooperation among authors.

Michael T. Lawton were the most productive authors, followed by Peter Vajkoczy, Ali Tayebi Meybodi, Gary K. Steinberg, and Jonathan J. Russin (Figure 4 and Table 4). It is not difficult to determine that Michael T. Lawton not only published the most works in the field of cerebral revascularization, but also collaborated the most closely. Figure 5A depicts a study of the author’s network of collaboration. Clusters of various hues represent organizations that work closely together. In the red cluster, Michael T. Lawton, Ali Tayebi Meybodi, and Arnau Benet are all from the United States, and the quantity of their papers accounts for the top 10 papers published overall. Michael T. Lawton and Arnau Benet are from the Department of Neurosurgery, St. Joseph’s Hospital, USA, and have a high level of collaboration in cerebral revascularization, with most of their articles published in Surgical Neurosurgery and World Neurosurgery (Gandhi et al., 2019; Hendricks et al., 2022). Peter Vajkoczy and Jan karl Burkhardt located in the yellow cluster are from Benevolence University Berlin and the University of Pennsylvania, respectively, who are closely associated in implantation of intracranial stents and angioplasty (Dengler, 2015; Lenga et al., 2019). Robert F. Spetzler and Laligam N. Sekhar of St. Joseph’s Hospital and the University of Washington, respectively, conduct cutting-edge research in the field of neurology intracranial aneurysms and are represented in the green cluster (Sekhar et al., 2018; Spetzler et al., 2019). Luca Regli and Fady T. Charbel are distinguished researchers in their respective specialties inside the blue and purple clusters, respectively. In the study of cerebral bypass surgery for intracranial arteriovenous malformations and intracranial aneurysms, they are closely related (Stapleton and Charbel, 2019; Staartjes et al., 2021). It is undeniable that most of the highly productive scholars in the field of cerebral revascularization are from American universities and research institutions, and they are more mature in the development and clinical operation of related technologies. Of course, this is also due to the country’s economic strength and the influence of science and technology. Therefore, the boundaries between scientific research should be abandoned, and scholars in the field of cerebral revascularization should be encouraged to strengthen the communication at the technical level.

**TABLE 4:** Authors with the highest number of publications and citations (top 10).

Rank	Count	Author	Rank	Count	Cited author
01	27	Lawton, Michael T	01	361	Sekhar, In
02	20	Vajkoczy, Peter	02	257	Fujimura, M
03	17	Meybodi, Ali Tayebi	03	247	Kuroda, S
04	16	Steinberg, Gary K	04	211	Sundt, Tm
05	12	Russin, Jonathan J	05	198	Lawton, M T
06	12	Burkhardt, Jan-karl	06	195	Barnett, Hjm
07	12	Ahn, Jae Sung	07	193	Houkin, K
08	12	Ravina K	08	183	Powers, Wj
09	11	Tulleken C	09	182	Suzuki, J
10	11	Charbel F	10	164	Spetzler, Rf

Author co-citation analysis is to determine the distance of research interest among authors by analyzing the frequency of papers published by different authors and simultaneously cited by authors of other papers. Using the VOSviewer software, we analyzed the top 10 most co-referenced and cited authors once again (Figure 5B and Table 4). 19 writers have had their works referenced more than 100 times, reflecting the high scientific value and international significance of their research. M. Fujimura, S. Kuroda, and a number of other researchers improved the prognosis of adult moyamoya disease by unblocking the STA-MCA bypass (Fujimura and Tominaga, 2015; Kuroda, 2020), primarily through middle cerebral artery anastomosis, thereby decreasing persistent intracranial angiogenic edema. In the green cluster, In. Sekhar and Tm. Sundt et al. successfully addressed the long-term prognosis of patients with skull base tumors and intracranial aneurysms by performing high-flow bypass surgery and aneurysm blocking of middle cerebral artery branch blood flow (Hage, 2019; Samadashvili et al., 2019). In addition, they examined research on cerebral revascularization for the treatment of ischemia, aneurysms and malignancies of the skull base. Within the blue cluster, Hjm. Barnett and S. Amin Hanjani utilized the superficial temporal artery (STA) as the primary donor channel for extracranial intracranial bypass surgery. It is stated that cerebral blood supply repair should be avoided in the case of subarachnoid hemorrhage after describing the pros and cons of these procedures (Zhang et al., 2002; Hage et al., 2015). Of course, the authors’ co-citation analysis can clearly reflect the closeness of their research directions and reveal their respective or co-representative research fields and research frontiers. Therefore, for research in the field of cerebral revascularization, we should advocate close communication between high co-citation authors, which can not only promote the maturity of research techniques in this field, but also stimulate more cutting-edge innovations. At the same time, this direct academic exchange between authors can further provide new therapeutic strategies for ischemic cerebrovascular diseases, thus making a significant contribution to the development of neurology.

### 3.4. Distribution of core journals

**FIGURE 6:**
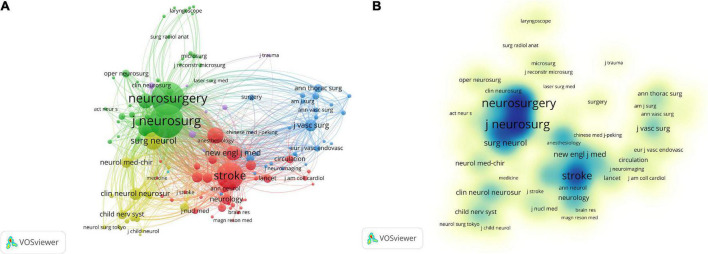
Analysis of co-cited journals. **(A)** Network diagram of co-cited journals based on VOSviewer visual map. **(B)** Visualization of the density of co-cited journals based on VOSviewer visual map. Different color clustering reflects the cooperation between co-cited journals.

The researchers have published papers on 169 journals, and we list the top 10 journals (Table 5). Among these journals, 12 journals published at least 15 articles related to cerebral revascularization. World Neurosurgery is the journal with the highest number of articles related to cerebral revascularization (111 articles), followed by Journal of Neurosurgery (72), Neurosurgery (71), Acta Neurochirurgica (49), and Stroke (29). After analyzing the data, around 50% of the publications are from the top 10 journals. The average impact factor (IF) of the top 10 journals is 3.80, and the average H-index is 130.1. Approximately 70% of these journals are published in the United States. A total of 39 journals have been cited more than 100 times. Co-citation analysis of journals is an organic way of linking a large number of various journals that are not externally connected, revealing the interdependence and interaction between journals. This feature can be used to determine the professional scope of certain journals and to help identify the core academic journals in a discipline. In Figures 6A, B, the top three journals jointly cited are Stroke, Neurosurgery, and Journal of Neurosurgery, which have been cited more than 8,000 times in total, and are also the three most distinguished journals in the field of neurosurgery. In addition, the analysis of the core journals in this field will help us more accurately grasp the main publication carriers in the field of cerebral revascularization. This will enable the general public or researchers in other disciplines to quickly grasp the first-hand information in the field of cerebral revascularization. This is of great significance for the development of the discipline and the integration of multidisciplinary exchanges.

**TABLE 5:** Top 10 journals.

Rank	Count	Journals	IF	H-index	Country
01	111	World Neurosurgery	2.21	101	United States
02	72	Journal of Neurosurgery	5.32	219	United States
03	71	Neurosurgery	5.15	207	United States
04	49	Acta Neurochirurgica	2.82	100	Austria
05	29	Stroke	7.82	330	United States
06	28	Operative Neurosurgery	2.67	26	United States
07	21	Neurosurgical Focus	3.64	102	United States
08	17	Neurosurgical Review	2.95	64	Germany
09	16	Journal of Clinical Neuroscience	2.12	85	United States
10	15	Journal of Stroke and Cerebrovascular Diseases	2.68	67	United Kingdom

### 3.5. Keyword and hot spot analysis

**FIGURE 7:**
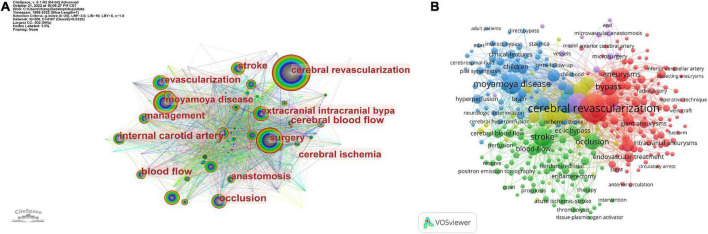
Keyword analysis. **(A)** Keyword network map based on Citespace visualization. Each node represents a keyword and each line represents the central cooperation relationship between keywords. **(B)** Keyword analysis based on the VOSviewer visual map. The clusters of different colors reflect the interrelationship between keywords.

A limited number of keywords can clearly describe the information about the main research objects, research questions, use of methods and research conclusion of scientific research achievements, and can reflect the research topics of scientific research achievements to a certain extent. As illustrated in Figure 7A and Table 6, the Citespace analysis reveals that 28 keywords appear more than 40 times. The top five of them are “cerebral revascularization,” “moyamoya disease,” “surgery,” “extracranial intracranial bypass,” and “occlusion.” “internal carotid artery” appears 86 times as the keyword with the most centrality, followed by “occlusion,” “revascularization,” “blood flow” and “stroke.” In Figure 7B, the keywords are essentially categorized into three distinct groups: red, blue, and green. The most important keywords in the red cluster are “cerebral revascularization,” “surgery,” “occlusion,” and “bypass.” This cluster focuses mostly on various surgical application approaches for cerebral revascularization. The most important keywords in the blue cluster are “revascularization,” “moyamoya disease,” and “disease.” By a comprehensive literature review, it is discovered that neurosurgical treatment of moyamoya disease is an effective method for curing the disease (Jang et al., 2017; Liu et al., 2017). The majority of the disease occurs at the end of the internal carotid artery, which is also the cerebral blood supply’s hub. Consequently, surgical treatment can restore cerebral blood circulation and slow the progression of the condition. The most important keywords in the green cluster are “extracranial intracranial bypass,” “stroke,” and “sta-mca bypass.” Extracranial intracranial bypass is an established surgical technique for cerebral revascularization, whose objective is to improve distal cerebral circulation, hence minimizing the damage caused by cerebral stroke (Thanapal et al., 2018). Although the construction of bypass necessitates the temporary blockage of receptor arteries, proper perioperative management reduces the risk of intraoperative cerebral ischemia associated with temporary vascular occlusion (Horn et al., 2008).

**TABLE 6:** Key words in publications and centrality (top 10).

Rank	Count	Centrality	Keywords	Rank	Centrality	Count	Keywords
01	408	0.07	Cerebral revascularization	01	0.12	86	Internal carotid artery
02	220	0.06	Moyamoya disease	02	0.1	113	Occlusion
03	207	0.06	Surgery	03	0.1	110	Revascularization
04	120	0.04	Extracranial intracranial bypa	04	0.1	67	Blood flow
05	113	0.1	Occlusion	05	0.09	90	Stroke
06	110	0.1	Revascularization	06	0.07	408	Cerebral revascularization
07	100	0.06	Superficial temporal artery	07	0.07	66	Anastomosis
08	90	0.06	Aneurysm	08	0.07	53	Cerebral blood flow
09	90	0.09	Stroke	09	0.07	42	Outcm
10	86	0.12	Internal carotid artery	10	0.07	32	Cerebral ischemia

**FIGURE 8:**
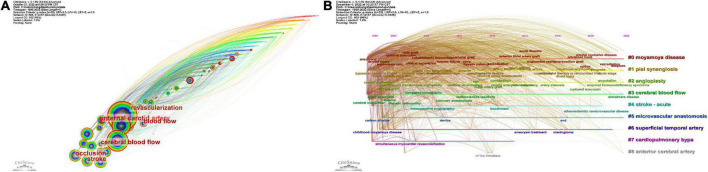
Visualization of keyword views. **(A)** Time zone view. **(B)** Timeline view.

Figures 8A, B depict the time zone view and timeline view of keywords, respectively. Table 7 displays the first nine clusters of related high-frequency words. The terms “moyamoya disease,” “Pial synangiosis,” “angioplasty,” “cerebral blood flow,” and “stroke-acute” appear in a number of articles pertaining to cerebral revascularization and also appear in big clusters in our computer analysis. Moyamoya disease is currently the main disease studied by numerous researchers in the field of cerebral revascularization (Fujimura et al., 2016). Pial synangiosis is a safe and long-lasting approach of cerebral revascularization, which is a viable treatment for adult moyamoya disease (Jecko et al., 2016). Angioplasty can enhance the blood flow of the external carotid artery (ECA), which includes the STA, and improve the cerebral blood flow of patients with cerebral vascular disease or cerebral vascular reconstruction failure treated by STA-MCA bypass (Leung et al., 2016). These investigations are based on the reconstruction of cerebral blood circulation, and neurosurgery is used to augment cerebral blood supply, which significantly improves the short-term effectiveness and long-term prognosis of stroke patients. Through keyword clustering analysis, we can accurately grasp the trend fluctuation of the importance of various keywords, and find out the attention status and trend of the topics reflected by these keywords in the current scientific research work, so that scholars can analyze and study the related topics again, which is conducive to highlighting key topics and grasping key research problems.

**TABLE 7:** Top 9 clusters of keywords.

Cluster ID	Size	Year	Label
#00	142	2009	Moyamoya disease
#01	107	2010	Pial synangiosis
#02	86	2010	Angioplasty
#03	66	2004	Cerebral blood flow
#04	29	2010	Stroke-acute
#05	27	2009	Microvascular anastomosis
#06	21	2011	Superficial temporal artery
#07	16	2003	Cardiopulmonary bypa
#08	8	2010	Anterior cerebral artery

Keywords burst can accurately reflect the new trend of research in this field in a period of time. The weight of major keywords is analyzed according to the order of keywords given in the literature and transformed into time series data to study the correlation between keywords from the perspective of time change, which provides a feature description method with similar distribution rule for scientific research topic analysis. Table 8 lists, in chronological order, the top 25 terms with the greatest citation growth. The intensity of the bursts reflects the intensity of oscillations in keyword frequency over this period. From 1999 to 2008, “internal carotid artery” and “giant aneurysm” were the top two keywords, while from 2009 to 2022, the top two keywords changed into “hyperperfusion” and “vascular disorder.” In contrast, the bursts of phrases such as “blood flow reactivity,” “acetazolamide test,” “surgical revascularization,” “indirect bypass,” and “synangiosis” were very brief. Therefore, these are likely the most pertinent phrases in the field of cerebrovascular disease at various times.

**TABLE 8:** Keywords with the strongest citation bursts (top 25).

Keywords	Year	Strength	Begin	End	1999–2022
Giant aneurysm	1999	7.87	1999	2008	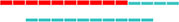
Cerebral ischemia	1999	4.44	1999	2005	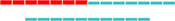
Saphenous vein graft	1999	4.32	1999	2008	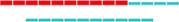
Acetazolamide test	1999	3.62	1999	2010	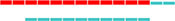
Internal carotid artery	1999	10.58	2000	2008	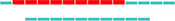
Occlusive disease	1999	3.87	2000	2008	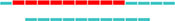
Blood flow reactivity	1999	3.58	2000	2004	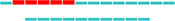
Balloon test occlusion	1999	4.57	2001	2003	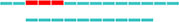
Cerebrovascular reserve capacity	1999	4.36	2001	2011	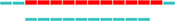
Angioplasty	1999	4.18	2003	2010	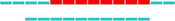
Technical note	1999	3.73	2003	2005	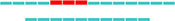
Carotid artery	1999	3.96	2004	2011	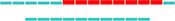
Carotid artery occlusion	1999	4.31	2005	2011	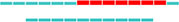
Randomized trial	1999	4.73	2008	2016	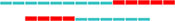
Pediatric moyamoya	1999	4.63	2009	2017	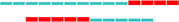
Endovascular treatment	1999	3.79	2011	2018	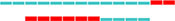
Brain ischemia	1999	3.64	2011	2015	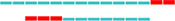
Hyperperfusion	1999	5	2014	2018	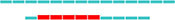
Outcm	1999	3.7	2014	2016	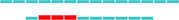
Surgical revascularization	1999	3.49	2016	2022	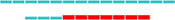
Indirect bypa	1999	3.66	2017	2022	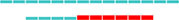
Synangiosis	1999	3.64	2018	2022	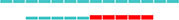
Vascular disorder	1999	4.92	2019	2022	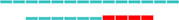
Cerebral bypa	1999	3.98	2019	2022	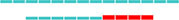
Sta-mca bypa	1999	3.91	2020	2022	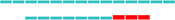

Red color represents the length of the burst year for this keyword in this time region and blue color represents the lack of burst years for that keyword in that time region.

In summary, we believe that the analysis of keywords in various disciplines should be done from the perspective of data mining and time series. According to the importance and centrality size of keywords in scientific achievements, the popularity of keywords is studied from two perspectives: numerical value and trend, respectively. This can provide decision-making techniques and solutions for problems related to the research field. In the field of cerebral revascularization, we should first focus on the major diseases “moyamoya disease,” “Pial synangiosis” and “stroke-acute.” Secondly, we should be proficient in the operation of cerebral revascularization techniques such as “extracranial intracranial bypass,” “angioplasty,” and “microvascular anastomosis.” In addition, the new direction of future research based on “hyperperfusion” and “vascular disorder” can provide a theoretical basis for in-depth research in the field of cerebral revascularization.

### 3.6. Distribution of cited articles and co-cited references

**FIGURE 9:**
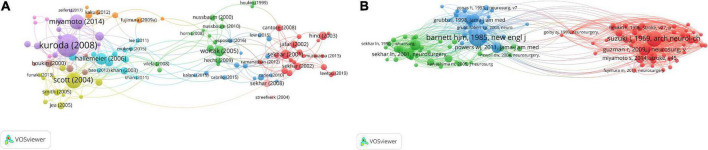
Analysis of cited articles and co-cited references. **(A)** Network diagram of cited articles based on VOSviewer visual map. **(B)** Network diagram of co-cited references based on VOSviewer visual map. Different color clustering reflects the close relationship between cited articles and co-cited references.

With the advancement of science and technology, it has become increasingly difficult to find the information most relevant to a scholar’s research from the vast amount of data. With the development of scientometrics, citation information is often listed in literature searches, so as to improve the efficiency of literature retrieval. 134 articles and 42 references were cited more than 30 times using VOSviewer analysis. The assessment of cited papers and co-cited references pertinent to investigations on cerebral revascularization is depicted in Figures 9A, B. William E. Boden, Satoshi Kuroda, and RM. Scott are the authors most frequently cited by academia (Table 9). The article “Niacin in Patients with Low HDL Cholesterol Levels Receiving Intensive Statin Therapy” written by William E. Boden was published in New England Journal of Medicine in 2011 and has been cited 1,925 times. Random assignment was used to assign 3,414 people to the niacin therapy group or the placebo group. Each day, the niacin group received at least 1,500 cc of niacin with delayed release. After 3 years of follow-up, the trial was terminated due to lack of efficacy. However, after 2 years of statistical analysis, it was determined that niacin could improve the risk index of atherosclerotic cerebrovascular disease, enhance the effect of cerebral revascularization, and reduce the mortality of cerebrovascular disease (Investigators et al., 2011). In addition, Japanese researcher Satosh Kuroda noted in a 2008 Lancet Neurology article titled “Moyamoya disease: current concepts and future perspectives” that extracranial intracranial artery bypass surgery includes anastomosis and indirect bypass between the STA and the middle cerebral artery (Kuroda and Houkin, 2008), which can help prevent further ischemic attacks and restore cerebral blood circulation (Miyamoto et al., 2014). In addition, research on cerebral revascularization is always evolving, and the works of these researchers are regularly acknowledged. Four of the top 10 most-cited literature were published in Stroke, while two were published in the two most-respected journals in the field of cerebral revascularization: New England Journal of Medicine and Lancet Neurology. At the same time, we should pay attention to the role of cited literature in the field of cerebral revascularization. These literature are scientific achievements with significant research prospects in this field. In addition, we should dig deeper into the co-citation links of the scientific literature, which can not only describe the current research status of the discipline, but also trace the dynamic development process of the discipline.

**TABLE 9:** Top cited articles (top 10).

Rank	Title	Authors	Citations	Journal	Year	DOI	IF
01	Niacin in patients with low HDL cholesterol levels receiving intensive statin therapy	Boden, William E	1925	New England Journal of Medicine	2011	DOI:10.1056/NEJMoa1107579	74.699
02	Moyamoya disease: Current concepts and future perspectives	Kuroda, Satoshi	656	Lancet Neurology	2008	DOI:10.1016/S1474-4422(08)70240-0	30.039
03	Long-term outcome in children with moyamoya syndrome after cranial revascularization by pial synangiosis	Scott, RM	368	Journal of Neurosurgery	2004	DOI:10.3171/ped.2004.100.2.0142	5.32
04	Effects of extracranial-intracranial bypass for patients with hemorrhagic moyamoya disease results of the Japan adult moyamoya trial	Miyamoto, Susumu	321	Stroke	2014	DOI:10.1161/STROKEAHA.113.004386	7.82
05	MRI-based selection for intra-arterial stroke therapy value of pretreatment diffusion-weighted imaging lesion volume in selecting patients with acute stroke who will benefit from early recanalization	Yoo, Albert J	244	Stroke	2009	DOI:10.1161/STROKEAHA.108.541656	7.82
06	Clinical features and outcome in North American adults with moyamoya phenomenon	Hallemeier, C.L	224	Stroke	2006	DOI:10.1161/01.STR.0000221787.70503.ca	7.82
07	Collateral growth and angiogenesis around cortical stroke	Wei, L	223	Stroke	2001	DOI:10.1161/hs0901.094282	7.82
08	Intraoperative control of extracranial-intracranial bypass patency by near-infrared indocyanine green videoangiography	Woitzik, J	191	Journal of Neurosurgery	2005	DOI:10.3171/jns.2005.102.4.0692	5.32
09	A systematic review of outcomes following staged and synchronous carotid endarterectomy and coronary artery bypass	Naylor, AR	187	European Journal of Vascular and Endovascular Surgery	2003	DOI:10.1053/ejvs.2002.1895	5.32
10	Cerebral revascularization using radial artery grafts for the treatment of complex intracranial aneurysms: Techniques and outcomes for 17 patients	Sekhar, LN	155	Neurosurgery	2001	DOI:10.1097/00006123-200109000-00023	5.15

## 4. Discussion

### 4.1. General information

In this study, we conducted a bibliometric analysis of the literature related to cerebral revascularization over a 20-year period and included 854 publications in the WOSCC database. The results indicate that research in the field of cerebral revascularization has increased significantly since 2008, and the number of published publications has stabilized since 2013, remaining between 40 and 50 each year. The United States has the biggest output and proportion in the field of cerebral revascularization, followed by Japan and China, according to an analysis of published countries. In the analysis of national centrality, the United States remains the first, followed by Japan, and the majority of European nations. This also demonstrates that the field of cerebral revascularization in neurosurgery in the United States is the most advanced in the world and has close ties to other nations. In the analysis of research institutes, it is discovered that the United States and Japan have the leading research institutes in the field of cerebral revascularization, for instance, the University of California, San Francisco, Stanford University, St. Joseph’s Hospital in Los Angeles, and Tohoku University. In the examination of the top 10 publications, however, the Capital Medical University of China ranks the third. Although the United States ranks the first globally in terms of country/institution and centrality analysis, it cannot dismiss the contributions of Asian nations such as Japan and China to cerebral revascularization research. All these demonstrate that the current international distribution of this research topic is uneven.

Michael T. Lawton is not just the most prolific author in the subject of cerebral revascularization, but also one of the five most-cited researchers. He has conducted extensive study on intracranial aneurysms and cerebrovascular abnormalities during his career in neurosurgery (Gandhi et al., 2019; Hendricks et al., 2022). For MCA surgical therapy for recurrent and resistant aneurysms, he presented the fourth generation of bypass technology (Burkhardt et al., 2018), M2-M2 Side-Side Reimplantation, to mend narrowed blood vessels. The left lateral fissure was opened by the left pterional approach, and it was determined that one branch of the lower and higher M2 trunks was near and parallel, with a diameter of approximately 2 mm, and was therefore suitable for lateral anastomosis (*in situ* anastomosis). The cerebral blood supply was restored by first anastomosing the ventral vascular wall and then constantly suturing the dorsal vascular wall using the two fixed point continuous suture method. The M2-M3 lateral anastomosis was not blocked, nor was the aneurysm of the M2 upper trunk visible. The anastomosis transferred the blood flow of the M2 lower trunk to the M2 upper trunk, and the M2 upper trunk’s blood supply was optimal. Simultaneously, he estimated that preoperative embolization could decrease the incidence rate of high-risk cerebral arteriovenous malformations (Lawton and Lang, 2019). In. Sekhar, who has the most cited articles in the field of cerebral revascularization, is also a highly influential author. In response to Dr. Ravina K’s publication on three vessel anastomosis (Sekhar et al., 2020), he convincingly argued that this technology can be utilized to treat bilateral cerebral vascular reconstruction. Peter Vajkoczy are also prominent researchers in the field of cerebral revascularization. Using STA-MCA bypass and minimally invasive cerebral sclerosis vascular anastomosis, he successfully reconstructed cerebral blood circulation in the treatment of moyamoya disease (Lucia et al., 2022). He considered that the STA-MCA bypass was preferable to the MIS-EDS system. When paired with direct bypass, STA-MCA bypass was suggested as an additional cerebral revascularization technology option. Due to their extensive clinical expertise, these authors enjoy a solid reputation in the field of cerebral revascularization research. In addition, the effective application of these cerebral revascularization techniques cannot be achieved without two important institutions: universities and hospitals. Among them, the primary stage of research and development is based on the continuous innovation of university teams. For example, the development of the three-vessel anastomosis technique was based on animal experiments by selecting Y-bypass and A3 or A4 blood vessels of two distal anterior cerebral arteries (ACA) for side-to-side anastomosis (Ravina and Russin, 2020). Of course, the maturity of difficult neurosurgical techniques was first based on the university platform. Finally, all these techniques will be applied to clinical practice, thus rapidly resolving the symptoms of neurological deficits in patients with acute cerebral ischemia. Thus, the high level of skill of academics and the research and development, university groups, and large hospitals play an important role in the field of cerebral revascularization.

In general, the impact factor of a journal is calculated in terms of years (Liu et al., 2016). The calculation formula is IF = (nk−1 + nk−2)/(Nk−1 + Nk−2), where “k” represents a specific year, Nk−1 + Nk−2 represents the number of articles published in the journal during the previous 2 years, and nk−1 + nk−2 represents the number of articles cited in the journal during the previous 2 years, i.e., IF. Typically, we believe that the journal’s influencing variables are directly tied to the quality of the articles. Stroke is the journal with the greatest impact factor in the ranking of published journals. Stroke, which was established in 1970, is the official journal of the American Stroke Association. It is a journal dedicated to cerebral circulation, and its primary acceptance fields include clinical and fundamental research in the field of cerebral circulation. Currently, the magazine is an extremely authoritative publication in the field of neurology. World Neurosurgery is the journal that contains the greatest number of publications on cerebral revascularization, which is published in the United States by Elsevier Company. It focuses mostly on research in clinical neurology surgery. In recent years, this journal’s impact factors have increased, indicating that neurosurgery’s effect is growing increasingly pervasive. In addition, the majority of journals, such as Journal of Neuroscience, Neuroscience, Operational Neuroscience, and Neurological Focus, belong to the category of professional disciplines. Some comprehensive journals, such as the Journal of Clinical Neuroscience and the Journal of Stroke and Cerebrovascular Diseases, have also published research on cerebral revascularization. Revascularization of the brain is a promising area of neurosurgical research. Numerous research publications in this topic have been published in various high-quality journals, which also suggests that this field has gained considerable attention from scholars worldwide. In addition, the publication of research in the field of cerebral revascularization in multidisciplinary publications is beneficial to attracting the attention of a greater number of researchers, thereby facilitating the expansion of interdisciplinary cooperation.

### 4.2. Hot spots and frontiers

Analysis of keyword co-occurrence permits us to comprehend the new hot spots and frontiers in a sector (Gao et al., 2021). In this study’s keyword analysis, we identified several significant research hot spots in the field of cerebral revascularization over the past two decades, including “cerebral revascularization,” “moyamoya disease,” “surgery,” “extracranial intracranial bypass,” “occlusion,” “revascularization,” “superficial temporal artery,” “aneurysm,” “stroke,” and “internal carotid artery” (Table 6). We subsequently reviewed the relevant literature and discovered that, in recent years (Gunawardena et al., 2019; Kan et al., 2021), “extracranial intracranial bypass” or “STA-MCA bypass” is the most important neurosurgical treatment in the field of cerebral revascularization, and the majority of them have significant effects on the treatment and prognosis of moyamoya disease, for instance, the safety of STA-MCA bypass in the treatment of moyamoya disease (Liu et al., 2017), the hemodynamic problems of STA-MCA bypass in the treatment of moyamoya disease (Jeffree and Stoodley, 2009), the significance of Color Doppler monitoring of STA-MCA bypass in the treatment of moyamoya disease (Kawamata et al., 2011; Feghali et al., 2019). However, we employed keyword clustering analysis to determine the tight association within the cerebral revascularization research field. # 00 Moyamoya disease is the most closely linked keyword in the overall research category, and it is also the most extensively researched disease in the subject of cerebral revascularization. The others are # 01 Pial synangiosis, # 02 Angioplasty, # 03 cerebral blood flow, # 04 Stroke-acute, # 05 Microvascular anastomosis, # 06 Superficial temporal artery, # 07 Cardiopulmonary bypa, and # 08 Anterior cerebral artery.

However, the acquisition of the aforementioned keywords requires additional keyword data, and the update frequency may not be optimal. Therefore, we examine the explosive situation of keywords over time using keyword bursts. “internal carotid artery” and “giant aneurysm” were the most popular study topics in the field of cerebral revascularization from 1999 to 2008. With the progression of time, “hyperperfusion” and “vascular disorder” have become the most prominent areas of study. Similarly, while “blood flow reactivity,” “acetazolamide test,” “surgical revascularization,” “indirect bypass,” and “synangiosis” are somewhat connected to cerebral revascularization, they lack a continuous explosive trend. In conclusion, we feel that hyperperfusion and vascular disorder will be the primary focus and direction of future research in this subject. The decline in neurological function after cerebral revascularization may be associated with transient over perfusion of the superficial temporal artery/middle cerebral artery bypass (Yang et al., 2018), and the timing and location of over perfusion are also indirect interference factors of post-operative neurological function defect (Mukerji et al., 2015). In ischemic cerebrovascular illness, cerebral hyperperfusion is a common consequence after superficial temporal artery/middle cerebral artery bypass (Tashiro et al., 2018). Despite opposition to the use of “low flow” in this bypass, the flow rate of perfusion has not been precisely specified (Kaku et al., 2012). Perioperative injection of edaravone, a free radical scavenger for adult moyamoya disease, can decrease the likelihood of cerebral vascular hyperperfusion and transitory neurological deficit related to hyperperfusion (Uchino et al., 2016). In addition, the incidence of cerebral hyperperfusion has been estimated to range from 0.3 to 1.2% (Meyers et al., 2000), and it is regarded as a complication after carotid endarterectomy. Notably, transcranial Doppler (TCD), the only clinical device that can adequately assess cerebral hyperperfusion, provides a cerebral blood flow monitoring technology (Feghali et al., 2019). In addition, a number of studies indicate that, when performing superficial temporal artery/middle cerebral artery bypass surgery in elderly patients with moyamoya disease (Kim et al., 2022; Tsunoda et al., 2022), we must focus on strict blood pressure control and reduce the harmful cascade reaction caused by over perfusion during cerebral revascularization. Based on the findings of this study, hyperperfusion and vascular disorder will become future focal points and new research avenues in this sector.

### 4.3. Limitations

There are some potential limitations to this study. First, the bibliometric data for this study were obtained solely from the WOSCC database; no search for relevant content on other research platforms was conducted. Second, other types of articles, such as corrections and retractions, were uncovered throughout the search process and may have indirectly influenced the outcomes of the analysis. Due to the novelty and cutting-edge nature of this type of research, it may be possible to overlook the most recent research findings when updating the articles.

## 5. Conclusion

This paper takes 1999–2022 as the time region. Firstly, the field of cerebral revascularization was analyzed from both qualitative and quantitative perspectives, and in-depth social network analysis and content structure analysis were performed on countries and institutions. In addition, this paper also discusses and analyzes the future research directions in the field of cerebral revascularization. Overall, the United States is the country with the largest number of publications and the highest proportion of institutions and authors in this research field, and the United States also accounts for the majority of journals. In addition, countries have a network of collaborative relationships, but the intensity and density of cooperation is not ideal. For example, there is a lack of collaborative communication between the US, Japan, and China. Therefore, we strongly urge that academic barriers should be removed between these major publishing countries and institutions in each country to enhance international communication and cooperation to promote the strong development of the field of cerebral revascularization research.

Through the content analysis of the literature, it is found that current research on the topic of cerebral revascularization is focused on cerebral revascularization, moyamoya disease, extracranial intracranial bypass, and occlusion. Meanwhile, hyperperfusion and vascular disorder may become a new research focus in this field in the future. In general, this study will greatly promote the development of the field of cerebral revascularization research and provide an objective basis for exploring new directions in this field of research in the future.

More specifically, numerous researchers have recognized the huge impact that hyperperfusion has on the disease itself after cerebral revascularization, and that vascular disorder is a major factor that exacerbates the progression of this disease. Based on this, researchers will devote more energy to valuable research directions.

In addition, this study can quickly help beginners to accurately understand the current research hot spots in the field, as well as the main research countries/institutions and leading scholars in the field. At the same time, this study will help researchers from other disciplines and the general public to gain a deeper understanding of the dynamic development of the field of cerebral revascularization, thus better promoting the integration between multiple disciplines.

## Data availability statement

The original contributions presented in this study are included in the article/Supplementary material, further inquiries can be directed to the corresponding author.

## Author contributions

DZ: conception and design of the research, data analysis, and manuscript writing. XL, NJ, and WC: screening study data. YH: supervising research and revising manuscripts. All authors contributed to the article and approved the submitted version.
