# Supplementary material for: Bibliometric and visual analysis of cerebral revascularization from 1999 to 2022
Source: Front Neurosci. 2023 Jan 9;16:1088448. doi: 10.3389/fnins.2022.1088448 (PMC9868924; doi:10.3389/fnins.2022.1088448)
Supplement: Supplementary file 1 [file Data_Sheet_1.docx]

Supplementary Material

# Supplementary Data

**Search Queries:**

# TS＝（“Cerebral Revascularization”OR“Revascularization, Cerebral”OR“Cerebral Microsurgical Revascularization”OR“Cerebral Microsurgical Revascularizations”OR“Revascularization, Cerebral Microsurgical”OR“Brain Revascularization”OR“Revascularization, Brain”OR“Microsurgical Revascularization, Cerebral”OR“Extracranial-Intracranial Arterial Bypass”OR“Arterial Bypass, Extracranial-Intracranial”OR“Arterial Bypasses, Extracranial-Intracranial”OR“Bypass, Extracranial-Intracranial Arterial”OR“Bypasses, Extracranial-Intracranial Arterial”OR“Extracranial Intracranial Arterial Bypass”OR“Extracranial-Intracranial Arterial Bypasses”OR“EC-IC Arterial Bypass”OR“Arterial Bypass, EC-IC”OR“Arteral Bypasses, EC-IC”OR“Bypass, EC-IC Arterial”OR“Bypasses, EC-IC Arterial”OR“EC IC Arterial Bypass”OR“EC-IC Arterial Bypasses”OR“STA-MCA Bypass”OR“Bypass, STA-MCA”OR“Bypasses, STA-MCA”OR“STA MCA Bypass”OR“STA-MCA Bypasses”)

**Timespan=1999-2022**
